# Co-Variation of Depressive Mood and Locomotor Dynamics Evaluated by Ecological Momentary Assessment in Healthy Humans

**DOI:** 10.1371/journal.pone.0074979

**Published:** 2013-09-13

**Authors:** Jinhyuk Kim, Toru Nakamura, Hiroe Kikuchi, Tsukasa Sasaki, Yoshiharu Yamamoto

**Affiliations:** 1 Graduate School of Education, the University of Tokyo, Tokyo, Japan; 2 Department of Psychosomatic Research, National Institute of Mental Health, National Center of Neurology and Psychiatry, Tokyo, Japan; Catholic University of Sacred Heart of Rome, Italy

## Abstract

Computerized ecological momentary assessment (EMA) is widely accepted as a “gold standard” method for capturing momentary symptoms repeatedly experienced in daily life. Although many studies have addressed the within-individual temporal variations in momentary symptoms compared with simultaneously measured external criteria, their concurrent associations, specifically with continuous physiological measures, have not been rigorously examined. Therefore, in the present study, we first examined the variations in momentary symptoms by validating the associations among self-reported symptoms measured simultaneously (depressive mood, anxious mood, and fatigue) and then investigated covariant properties between the symptoms (especially, depressive mood) and local statistics of locomotor activity as the external objective criteria obtained continuously. Healthy subjects (*N* = 85) from three different populations (adolescents, undergraduates, and office workers) wore a watch-type computer device equipped with EMA software for recording the momentary symptoms experienced by the subjects. Locomotor activity data were also continuously obtained by using an actigraph built into the device. Multilevel modeling analysis confirmed convergent associations by showing positive correlations among momentary symptoms. The increased intermittency of locomotor activity, characterized by a combination of reduced activity with occasional bursts, appeared concurrently with the worsening of depressive mood. Further, this association remained statistically unchanged across groups regardless of group differences in age, lifestyle, and occupation. These results indicate that the temporal variations in the momentary symptoms are not random but reflect the underlying changes in psychophysiological variables in daily life. In addition, our findings on the concurrent changes in depressive mood and locomotor activity may contribute to the continuous estimation of changes in depressive mood and early detection of depressive disorders.

## Introduction

Ecological momentary assessment (EMA) is a method of acquiring self-reported information about a person’s subjective symptoms at the moment of recording and is used continuously in most cases to record instantaneous states of feeling without recall bias [1,2]. A paper-and-pencil diary was initially used for EMA, but it has been reported that the diary has the disadvantage of delayed data entry or even forward entry of data (i.e., “faked compliance”) [3]. Therefore, computerized EMA, which employs a computer as an electronic diary (ED), has been developed to avoid faked compliance; in this method, the input time is automatically registered in the device. Symptom diaries derived from computerized EMA are now generally regarded as the “gold standard” in the fields of psychiatric/psychosomatic medicine and has recently attracted increasing attention as an essential component for healthcare monitoring systems based on the information and communication technology (ICT) [4,5].

EMA scores for, e.g., depressive mood and fatigue, which are the key symptoms of depression [6] studied mainly in this paper, inevitably fluctuate because of their repetitive nature. Characteristics of these fluctuations have been studied by examining the relationships among simultaneously measured self-reported symptoms [7,8,9]. Many studies have also investigated that the fluctuations in psychological states are associated with those in objective measures sampled momentarily. For example, psychological stress is reported to be concurrently associated with cardiovascular parameters from blood pressures [10,11,12] and salivary cortisol [13,14,15,16]. Also, the co-variations between pulmonary functions tested by a spirometer and positive/negative affect in patients with asthma were reported [17]. In addition, some studies demonstrated that addictive behaviors such as smoking [18,19] and alcohol consumption [20,21] are related to the fluctuations in psychological states, e.g., positive/ negative affect and craving. Furthermore, the associations of momentary psychological states with self-reported physical activity were demonstrated [22,23].

To our knowledge, however, whether EMA scores co-vary with other symptoms evaluated simultaneously and with locomotor activity (habitual physical activity) measured continuously and un-obstructively, which is an important hypothesis for the interpretation of fluctuations of EMA scores, has not been rigorously tested. Therefore, the primary aim of this study was to test this hypothesis for groups of healthy subjects in two ways: evaluating (null) convergent associations among self-reported mood and fatigue scores and concurrent associations of the self-reported scores with external criteria provided by objective measures of locomotor activity, as described below.

It is considered that momentary micro-fluctuations in behavioral data, specifically those in locomotor activity capturing bodily acceleration counts in a continuous fashion, reflect the dynamics of systems organizing human behavior and can be used to probe behavioral disorders, including mental illnesses [24,25,26,27,28,29,30]. Indeed, altered locomotor activity is one of the cardinal signs of psychiatric disorders and included in their diagnostic criteria [6]. For example, major depressive disorders are known to be characterized by the presence of symptoms associated with behavioral alterations, such as diminished physical activity, loss of energy, psychomotor retardation or agitation, and sleep disturbances [6]. In order to quantitatively evaluate such alterations, several actigraphic studies have been conducted in depression patients by showing the significant decrease in activity levels during daytime and disruption of the circadian rhythm [24,25,26]. Furthermore, we recently found robust statistical properties of long-term (>7 days) locomotor activity altered in patients with major depressive disorders but not in healthy controls, reflecting increased intermittent bursts in the activity counts, characterized by reduced activity levels associated with occasional bursts of locomotor activity [31,32]. Therefore, the quantitative and objective evaluation of the levels and/or the intermittency of locomotor activity could provide appropriate behavioral measures capable of probing alterations in patients’ depressive mood and/or physical symptoms in daily life.

While our recent finding is thought to be important because it may lead to an objective evaluation of depressive disorders, whether changes in depressive mood are “momentarily” associated with such behavioral alterations probed by statistical properties of locomotor (or habitual physical) activity remains unknown. Thus, the secondary aim of this study was to search for the robust underlying associations between momentary depressive mood using EMA and local statistical properties of locomotor activity in healthy humans, regardless of group differences in age, lifestyle, and occupation by evaluating them with cross validation among various groups of subjects. Showing such associations is considered to be important because locomotor activity can be measured in a truly continuous manner, and finding objective correlates of momentary mood scores can compensate for a known drawback of EMA that the data based on sparse sampling may not provide contiguous data despite high levels of respondent load [33,34,35]. The continuous estimation of changes in depressive mood from locomotor activity might also help to detect the worsening of depressive mood even in healthy individuals at the beginning of pathogenic processes to depressive disorders.

## Results

### Recording profiles

The mean compliance rate, which is defined as the percentage of the number of completed recordings within 30 min from the first beep signal against the total number of time-based EMA during the study period, was 94.54% (SD = 5.96), and the mean number of recordings was 5.40 (SD = 1.58) per day for the adolescents [the mean number of event-based EMA recordings was 1.19 (SD = 0.88) per day for this group]; for the undergraduates, they were 92.03% (SD = 8.57) and 8.98 (SD = 1.43), respectively, and for the office workers, they were 95.40% (SD = 4.54) and 6.05 (SD = 4.49), respectively. The compliance rate was considerably high for all groups without significant differences (ANOVA, *F*_2,82_ = 1.92, *p* = 0.15). The mean scores of the three symptoms and the three local statistics of locomotor activity are summarized in Table 1.

**Table 1:** Statistics of self-reported symptoms and local statistics of locomotor activity. LA: locomotor activity, *SD*: standard deviation. ** and * indicate significant group difference at *p* < 0.01 and *p* < 0.05, respectively.

	Mean(SD)				
	All	Adolescents (G1)	Undergraduates (G2)	Office workers (G3)	Multiple comparison
**Self-reported symptoms**					
Depressive mood	39.93 (10.57)	40.00 (12.09)	40.31 (9.62)	39.34 (10.13)	
Anxious mood	31.54 (19.25)	33.81 (21.67)	31.40 (18.09)	28.88 (17.90)	
Fatigue	44.47 (18.43)	45.54 (19.04)	47.42 (18.18)	39.33 (17.65)	
**Local statistics of LA**					
Mean	144.44 (22.31)**	152.76 (17.97)	149.59 (22.00)	127.38 (18.72)	G3 < G1, G2
Skewness	0.04 (0.27)**	−0.11 (0.22)	0.11 (0.28)	0.14 (0.22)	G1 < G2, G3
Detrended skewness	0.06 (0.23)*	−0.03 (0.20)	0.08 (0.25)	0.14 (0.21)	G1 < G3

Note that all local statistics of LA were evaluated using a 60-min time frame centered around EMA recordings.

### Convergent associations among symptoms

Depressive mood showed significant positive correlation with fatigue (r = 0.26, *p* < 0.01) and anxious mood (r = 0.39, *p* < 0.01) for the data sets sampled from all groups. The correlation between fatigue and anxious mood was also positive and significant (r = 0.18, *p* < 0.01). These relations were consistent across groups (Table 2), indicating that all subjects properly performed the EMA recordings. The results also suggest the convergent validity of self-reported symptoms by EMA.

**Table 2:** Convergent associations (correlations) among self-reported mood and fatigue scores.

	depressive vs. anxious mood	depressive mood vs. fatigue	anxious mood vs. fatigue
All	0.39*	0.26*	0.18*
Adolescents	0.32*	0.25*	0.17*
Undergraduates	0.39*	0.38*	0.27*
Office workers	0.60*	0.25*	0.19*

* *p* < 0.01.

### Concurrent associations of depressive mood with local statistics of locomotor activity

#### Univariate multilevel modeling

**Figure 1:**
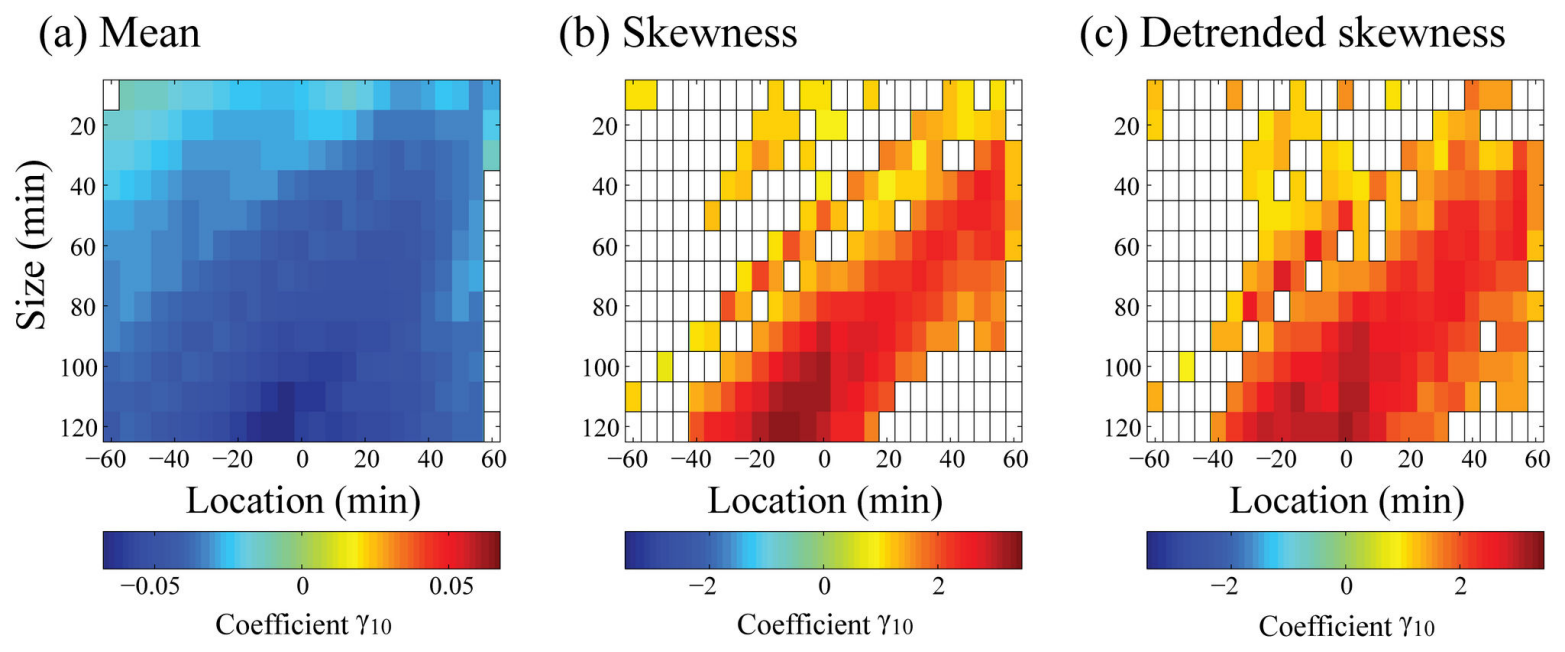
Dependency of univariate model coefficients for depressive mood on different time frames. The estimated values of the univariate model coefficient for depressive mood scores are shown in a colored matrix form consisting of 25 rows (different locations) and 12 columns (different sizes). Each grid cell indicates some specific location and size of a time frame used for calculating the local statistics of locomotor activity. A color in each cell represents the value of the model coefficient (γ_10_) of the predictors: (a) Mean, (b) Skewness, and (c) Detrended skewness. False discovery rate with a *q* value of 0.05 was used as the multiple comparison adjustment. Only significant cases are shown by colors. Color bars indicate the value of the model coefficient. Note that the univariate model used for the analysis is as follows; Depressive mood score_*ij*_ = γ_00_ + γ_10_ (Local statistics of locomotor activity_*ij*_) + ζ_0i_ + ε_*ij*_ (see Results for details).

The univariate multilevel analysis revealed simple correlations between the depressive mood scores and the local statistics (Figure 1). The mean and skewness as well as the detrended skewness showed significant correlations (FDR, *q* = 0.05). However, such relations depended significantly on the size and location of the time frame, especially for skewness (Figure 1). The mean levels of locomotor activity were negatively associated with depressive mood over a wide range of locations (60 min before to 55 min after) around the EMA recordings, and the associations were not significantly affected by the size of the time frame (data length 120–10 points) [Figure 1(a)]. This indicates that activity levels tend to be low with a higher level of depressive mood, and this relationship is consistent and robust over a long period. On the other hand, skewness (also detrended skewness) exhibited positive correlations in a limited range centered around the EMA recordings (e.g., from location −15 to 55 min with size 60 points for skewness, and −25 to 60 min for detrended skewness) [Figure 1(b) (c)]. This implies that depressive mood is associated with intermittent patterns of locomotor activity even in healthy populations, and this relationship appears instantaneously with the worsening of depressive mood. However, such a relationship disappeared with a small size of the time frame, which is partly attributed to the lack of statistical stability of skewness. Considering these results, we used the time frame centered around the EMA recordings with a size of 60 min while constructing the best-fitting model for depressive mood and examined the effects of the choice of time frame. For the univariate modeling, the coefficient of each predictor was fixed across the subjects because the inclusion of the random effect did not have a large effect on the results.

#### Determination of best-fitting model

Of all multilevel models evaluated, a model consisting of local mean and detrended skewness as the predictors was selected as the best-fitting model to describe the variations of depressive mood scores, where the predictor “mean” and intercept had a random effect. The best-fitting model is as follows: Depressive mood scores_*ij*_ = γ_00_ + γ_10_ (Mean_*ij*_) + γ_20_ (Detrended skewness_*ij*_) + ζ_0i_ + ζ_1i_ (Mean_*ij*_) + ε_*ij*_, where the Depressive mood scores_*ij*_ indicates a score of depressive mood at the *j*th recording for the *i*th subject; Mean_*ij*_ and Detrended skewness_*ij*_ are the local mean and detrended skewness of locomotor activity corresponding to the *j*th EMA recording (depressive mood score) for the *i*th subject, respectively; γ_00_ is the average intercept across all subjects; γ_10_ and γ_20_ are the average regression slopes across all subjects for each predictor; the random terms, ζ_0i_ and ζ_1i_, are the between-person residuals; and ε_*ij*_ is the within-person residual. As expected from the results of univariate multilevel analysis, the coefficient of both local statistics was significant (Table 3), with a negative value (−0.04 ± 0.01, *p* < 0.01) for the mean and a positive value (1.40 ± 0.69, *p* = 0.04) for the detrended skewness.

**Table 3:** Associations of depressive mood with local statistics of locomotor activity.

	Coefficient(*SE*)	*F* value	*p* value
***Alldataset***			
**Intercept** (γ_00_)	45.74 (2.40)		<0.01
**Coefficient of the local mean** (γ_10_)	−0.04 (0.01)	F_1,1386_ = 9.35	<0.01
**Coefficient of the local detrended skewness** (γ_20_)	1.40 (0.69)	F_1,1386_ = 4.18	0.04
***Crossvalidationacrossthethreegroups***			
**Intercept** (γ_00_)		*F*_1,82_ = 0.08	0.92
Adolescents	47.28 (3.95)		<0.01
Undergraduates	45.25 (4.30)		<0.01
Office workers	45.24 (4.20)		<0.01
**Coefficient of the local mean** (γ_10_)		F_1,1382_ = 0.07	0.94
Adolescents	−0.05 (0.02)		0.03
Undergraduates	−0.04 (0.02)		0.14
Office workers	−0.04 (0.02)		0.07
**Coefficient of the local detrended skewness** (γ_20_)		F_1,1382_ = 2.88	0.06
Adolescents	2.49 (0.99)		0.01
Undergraduates	2.26 (1.42)		0.11
Office workers	−1.24 (1.29)		0.34

*SE*: standard errors. The best-fitting model is as follows: Depressive mood scores_*ij*_ = γ_00_ + γ_10_ (Mean_*ij*_) + γ_20_ (Detrended skewness_*ij*_) + ζ_0i_ + ζ_1i_ (Mean_*ij*_) + ε_*ij*_. For cross validation, we assumed the coefficients (i.e., slopes) γ_10_ and γ_20_ are different across groups.

Although the best-fitting model marked the minimum AIC value, a model incorporating local mean with a random effect (F_1,1386_ = 9.80, *p* < 0.01) and skewness with a fixed effect (F_1,1386_ = 3.20, *p* = 0.07) was equally well-fitted to the data without any significant difference from the best-fitting model (see Table 4 for details). The common feature represented in these models is the intermittency of locomotor activity, characterized by a combination of reduced activity with occasional bursts of locomotor activity.

**Table 4:** Statistics of goodness-of-fit of best-fitting model and equally well-fitting models.

	Coefficient(*SE*)	*F* value	*p* value	AIC	*p* value*
**Best-fitting model**				11960.8	
Intercept	45.74 (2.40)		<0.01		
Coefficient of local statistics of locomotor activity					
Mean	−0.04 (0.01)	F_1,1386_ = 9.35	<0.01		
Detrended skewness	1.40 (0.69)	F_1,1386_ = 4.18	0.04		
**Well-fitting model**				11961.8	0.86
Intercept	45.92 (2.40)		<0.01		
Coefficient of local statistics of locomotor activity					
Mean	−0.04 (0.01)	F_1,1386_ = 9.80	<0.01		
Skewness	1.21 (0.68)	F_1,1386_ = 3.20	0.07		

AIC: Akaike information criteria. * *p* value for the change in AIC between the best-fitting model and each equally well-fitting model evaluated using the *z*-test.

#### Dependency on size and location

**Figure 2:**
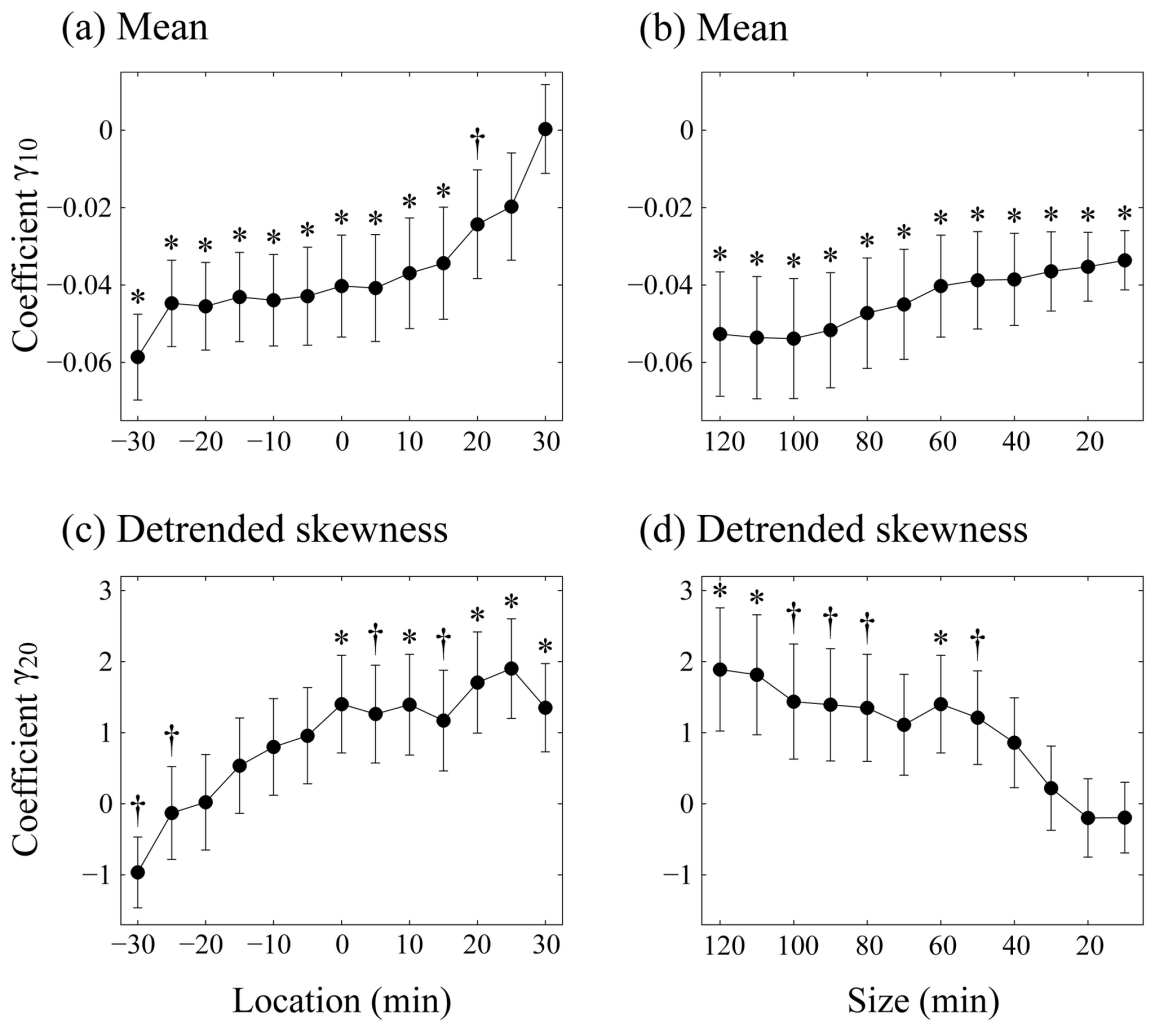
Dependency of best-fitting model coefficients for depressive mood on different time frames. The estimate of the model coefficient of the predictor: (a) Mean (γ_10_) and (c) Detrended skewness (γ_20_) of the best-fitting model for depressive mood scores (Depressive mood scores_*ij*_ = γ_00_ + γ_10_ (Mean_*ij*_) + γ_20_ (Detrended skewness_*ij*_) + ζ_0i_ + ζ_1i_ (Mean_*ij*_) + ε_*ij*_) as a function of the location. In panels (a) and (c), the size of the time frame was fixed at 60 min. The same is shown in panels (b) and (d), except for the dependency on the size of the time frame. For evaluation, the location of the time frame was fixed at 0 min; thus, the frame was centered around the EMA recordings. Error bars indicate standard error of the coefficients. The asterisk and dagger indicate significant cases at the 0.05 and 0.10, respectively.

We demonstrate the robustness of our model with regard to the choice of the size and location of the time frame by showing the dependency of the model coefficients on this choice (Figure 2). The coefficient of the “mean” was consistently significant with negative values (between −0.06 and −0.04) over a broader range (from location −30 to 20 min with size 60 points) [Figure 2 (a)]. Furthermore, this association was also robust against variation in sizes (120–10 points) [Figure 2 (b)]. In the case of “detrended skewness,” the significant positive coefficients were localized in a limited area around the EMA recordings (from 0 to 30 min with 60 points) [Figure 2 (c)], and this association was confirmed only at a larger size of the frame (data length > 50 points) [Figure 2 (d)].

### Cross validation for association of depressive mood with local statistics of locomotor activity

To examine the cross validation for the associations of depressive mood scores with local statistics of locomotor activity across the three groups, we considered one categorical variable representing “group.” There was no significant difference in the coefficient for either predictor (Table 3) among the groups, which supports the cross validity of our results. However, careful investigation of this model demonstrated that the inclusion of the categorical variable and the separate evaluation of the best-fitting model for each group led to insignificant model coefficients, except for the adolescents. This is caused by the difference in the sample size.

## Discussion

In this study, we investigated the nature of within-individual temporal variations in self-reported symptoms assessed using the EMA technique. EMA is known to be a powerful tool to overcome retrospective biases existing in recall-based self-reports and thus has recently attracted massive attention [1,2]. Many studies have demonstrated the strength of EMA in terms of the minimization of recall biases [36,37,38] and the correlation with other objective measures [10,11,12,13,14,15,16,17]. However, the concurrent associations of self-reported symptoms by EMA with objective real-time measures were not rigorously examined. Therefore, we investigated such associations between self-reported symptoms, especially depressive mood, and locomotor activity data, which can be measured in a truly continuous fashion. We then found the robust (against differences in groups of healthy subjects and the methods of EMA) within-individual temporal associations between fluctuations in depressive mood in daily life and the patterns of locomotor activity. We believe this finding could provide a novel insight into the psychophysiological correlates of “variations” of EMA recordings and a possibility to develop a continuous monitor for changes in depressive mood from locomotor activity, as discussed below.

### Validity of EMA

We confirmed the concurrent association of depressive mood with alterations in locomotor activity and the convergent associations among self-reported symptoms recorded simultaneously. In addition, we demonstrated the consistency and robustness of such associations across populations with different age groups, lifestyles, occupations, and so on and proved the existence of cross validity. These verifications provide strong evidence that the within-individual temporal variations in self-reported symptoms measured by EMA are not merely random but may partially originate from and reflect the alterations in psychophysiological states in daily life.

Our systematic approach to modeling the temporal variations in depressive mood scores using the local statistics of locomotor activity for the first time revealed the “momentary” associations of the depressive mood with the levels and patterns of locomotor activity. The intermittent bursts of locomotor activity were important for describing depressive mood even in healthy subjects, which is consistent with previous studies on major depressive disorders [31,32]. Considering the existence of the cross validity in these associations, we suggest that the present relations would generally hold for a wider range of populations. Whether this could be further extrapolated to patients suffering from depressive disorders would be the next important challenge.

The associations between depressive mood and alterations in the levels and patterns of locomotor activity have been shown in epidemiological samples [39,40,41,42,43] and clinical depression [31,32], but the underlying mechanism is unknown. Although the reasons for the covariant associations between depressive mood scores and behavioral alternations are still unclear, the present analyses of data with high temporal resolution clarify the mechanism, at least partially. As shown in Figure 1, there is less or virtually no association in the case of skewness measures reflecting intermittent activity patterns (shown in the upper left corner of the size vs. location plots). This implies that the association of the higher order statistics of locomotor activity data, even when recorded just before the EMA recordings, with the subsequent depressive mood scores is weak. Thus, it is most unlikely that behavioral alteration precedes momentary mood change, which is supported by higher coefficients for the detrended skewness of the best-fitting model at positive or posterior temporal locations [Figure 2 (c)]. The temporal characteristics of the covariant associations indicate that the behavioral alteration begins to be concurrent with mood change. This concurrency suggests that in addition to a possible effect of variations in depressive mood (or more instantaneous *feeling*) on the subsequent behavior, there is a more direct neurophysiological link between the changes in body state and (depressive) feeling, as proposed by Damasio [44]. On the other hand, the mean activity levels were associated with depressive mood over relatively longer time scales.

### Prospective for continuous monitoring of symptoms

Revealing the psychophysiological origin of the temporal variations in self-reported symptoms in terms of behavioral alterations is considered to be highly significant. EMA is known to have the critical drawback of sparse sampling over time; the number of data points recorded in a day is limited. In contrast, locomotor activity can be measured in a truly continuous fashion using a simple device. Therefore, our findings concerning the existence of psychobehavioral correlates between EMA recordings and behavioral alterations might be able to compensate for this drawback and contribute to the continuous estimation of changes in depressive mood in daily life, leading to the early detection or novel treatment of depressive disorders.

### Limitations

Several limitations should be noted. Our populations were limited to healthy subjects with different gender and age distributions. Another limitation is the inconsistency in the EMA protocols among groups leading to differences in the number of recordings and the time of day. Therefore, careful consideration is needed for generalizing our findings to other populations, especially patients with psychiatric disorders such as depression. Also, the model coefficients of the local statistics for the depressive mood scores were not consistently significant in all groups, which is considered to be caused by the small sample size. Therefore, further studies with a large number of subjects and data points would be necessary to rigorously examine cross validity. Finally, other local statistics of locomotor activity that can capture the intermittency in locomotor activity more robustly, such as entropy-type nonlinear statistics, should be considered.

## Conclusion

This is the first study providing an insight into the psychophysiological origin of within-individual temporal variations in momentary symptoms assessed by EMA by evaluating co-variant properties with continuous locomotor activity as the external criteria. We confirmed the robust concurrent associations between depressive mood and behavioral alterations and the convergent associations among self-reported symptoms. These findings indicate that temporal variations in momentary symptoms are not random but reflect changes in the underlying psychophysiological variables, represented as changes in the statistical properties of locomotor activity. We believe that our finding on the concurrent changes in depressive mood and locomotor activity may contribute to the continuous estimation of changes in depressive mood and early detection of depressive disorders.

## Methods

### Ethics statement

All subjects were given a thorough explanation of the purposes and potential risks of the study by well-trained researchers; then, the subjects signed an institutionally approved informed consent form. A written consent was also obtained from the adolescent subjects’ parents. The study was approved by the research ethics committee of the Graduate School of Education, the University of Tokyo.

### Subjects

A total of 113 healthy subjects were enrolled in this study; 35 were adolescents, 44 were undergraduates, and 34 were office workers. The adolescents were junior high school students in the secondary school attached to the Faculty of Education of the University of Tokyo. The undergraduates were recruited from the Department of Physical and Health Education at the University of Tokyo. Full-time employees at the University of Tokyo were recruited as the office worker subjects.

Of the 113 subjects, 28 were excluded from our analysis because their data were not measured properly owing to faults and/or subject error encountered while using the devices. We finally obtained data from 85 subjects consisting of 30 adolescents [4 males (M)/26 females (F), 13.6 ± 0.5 years of age (yrs)], 31 undergraduates (25M/6F, 21.6 ± 2.3 yrs), and 24 office workers (24M/0F, 41.0 ± 9.2 yrs).

### Materials, procedures, and measurements

#### Ecological momentary assessment

In this study, we used the EMA technique to examine momentary symptoms. The approach allows researchers to address subjects’ behavior, psychological states, and physiological reactions at multiple time points as they are experienced in daily life. Collecting data in natural settings can enhance the validity of measurements, thus avoiding the pitfalls of retrospective recall, which highly distort self-report data collection [1]. In the present study, we measured momentary symptoms during the study period using the EMA technique with the following procedures.

**Figure 3:**
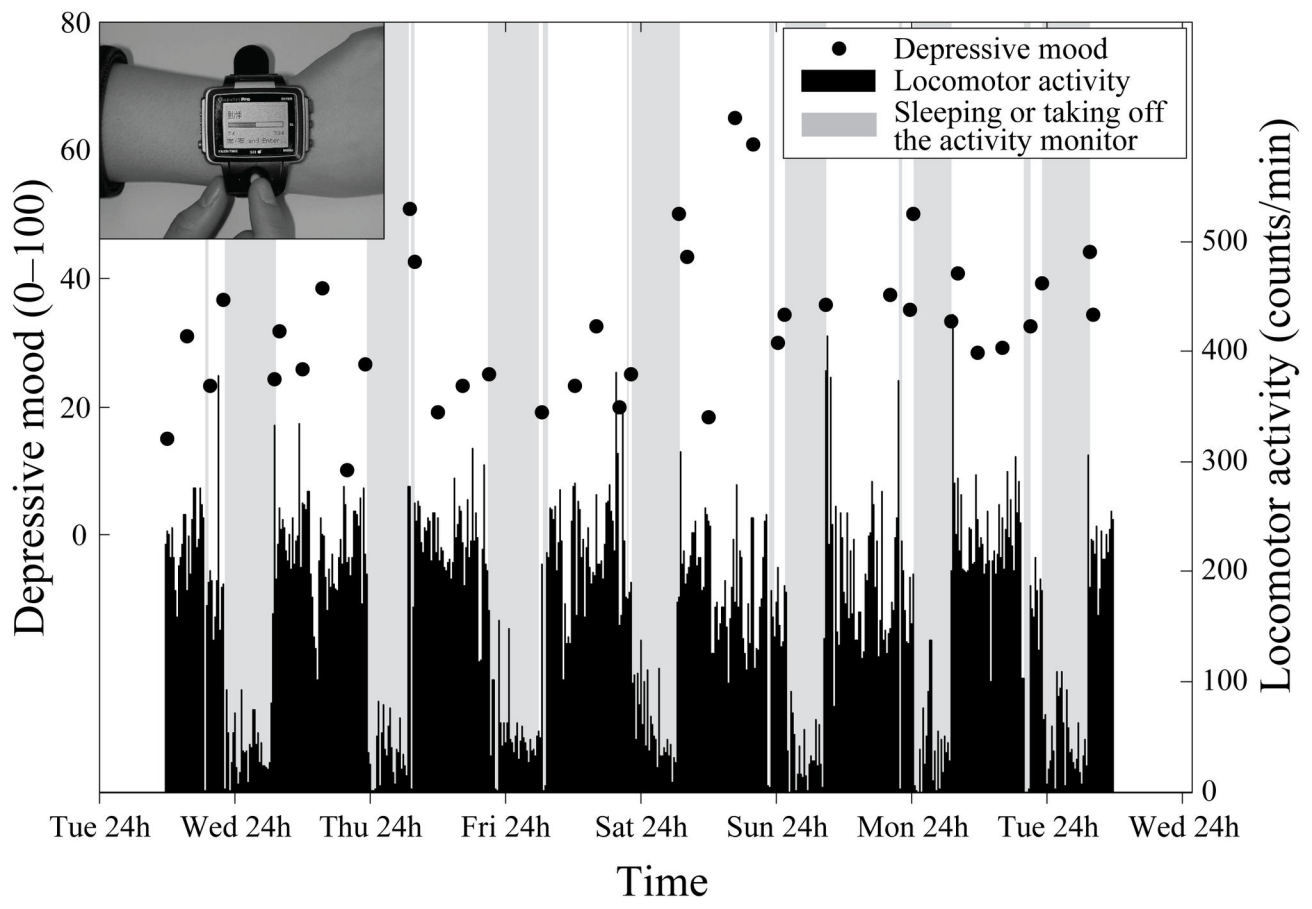
Fluctuation of depressive mood and locomotor activity in an office worker. Filled circles indicate depressive mood scores (y-axis on the left side) recorded by ecological momentary assessment (EMA) over seven consecutive days. The inset in the upper left represents the watch-type activity monitor equipped with EMA software. The locomotor activity measured simultaneously (zero-crossing counts within every 1 min time interval) is also shown (y-axis on the right side). The periods shaded in gray are times during which the subject was sleeping or had taken off the device.

A watch-type computer (Ruputer, ECOLOG, 42 g; Seiko Instruments Inc., Tokyo, Japan) [45] was used as the ED to record momentary symptoms. This device has a 20 mm × 30 mm screen and a joystick and buttons as input devices (Figure 3). The subjects were provided all appropriate instructions and also given manuals on how to use the device before the beginning of the study period; in addition, the subjects manipulated the device as a practice exercise until they became accustomed to it.

Adolescents: This group was instructed to complete the EMA questionnaires at randomly selected times within plus or minus 10 min of the predefined times (12:50, 16:30, and 20: 00) over seven consecutive days. The subjects were prompted to complete the questionnaires by a beep signal. If they could not enter the recordings when the device beeped, they were allowed to postpone the recordings twice for 15 min each time. If the recordings were not performed within 30 min, the corresponding EMA questionnaires were canceled.

Undergraduates: The EMA questionnaires were recorded over two consecutive days. The subjects were prompted by a beep signal to complete the EMA questionnaires on average every two hours during their waking period. The signals were programmed to occur randomly within intervals ±12 min of the predefined times (8: 00, 10: 00, 12: 00,…). The subjects were allowed to postpone the recordings twice for 10 min each time. If the recordings were not performed within 20 min, the corresponding questionnaires were canceled. The first experimental day of the week was randomly chosen, regardless of weekdays and weekends, so that the study periods can cover all possible day-conditions.

Office workers: EMA questionnaires were recorded on average every four hours during their waking period, over seven consecutive days. The signals were programmed to occur randomly within intervals ± 24 min of the predefined times (8: 00, 12: 00, 16: 00,…). The subjects were allowed to postpone the recordings twice for 10 min each time. If the recordings were not made within 20 min, they were canceled.

In addition, all subjects were asked to record the EMA questionnaires when they woke up and before they went to bed by choosing “waking up” or “going to bed,” from the menu. The selection of the latter item suspended the prompt for recordings until the former item was selected so that to the subjects’ sleep is not disturbed. Furthermore, they were instructed to remove the device before bathing, showering, or any other activity likely to damage the device. When they removed or wore the device, they were requested to select “taking off” or “putting on,” respectively, from the menu as well as the reason for removing the device. The adolescents were asked to conduct EMA recordings (i.e., event-based EMA [1]) in addition to the scheduled EMA (i.e., time-based EMA [1]) when they felt bad or had physical symptoms, such as severe fatigue, headache, or stomachache. Note that these data were analyzed without distinguishing between the two types of EMA because we obtained the consistent results whether such data points were included or not.

The EMA questionnaires assessed fatigue and mood states. The recording times were automatically registered in the device. Fatigue intensity and mood states were rated using a visual analog scale from 0 to 100, which was displayed on the device screen. The EMA questionnaires of momentary symptoms were randomly ordered, and each of them was displayed one at a time using a visual analog scale; the anchor words “none” and “most intense” were displayed at the respective ends of the scale. The subjects adjusted the length of the scale by manipulating the joystick of the device so that it corresponded to their subjective symptoms. Owing to the limited display resolution, the 0–100 scale for the intensity of subjective symptoms increased at 5-point intervals. Fatigue intensity was assessed by instructing the subjects to score their current fatigue level by presenting a single question with the adjective “fatigued.” Mood states were rated using the Depression and Anxiety Mood Scale (DAMS) [46], which was developed to measure anxious and depressive moods as separately as possible. The DAMS comprises nine adjectives representing different mood states: “vigorous,” “gloomy,” “concerned,” “happy,” “unpleasant,” “anxious,” “cheerful,” “depressed,” and “worried.” From these items, anxious mood (sum of “concerned,” “anxious,” and “worried” scores), positive mood (sum of “vigorous,” “happy,” and “cheerful” scores), and negative mood (sum of “gloomy,” “unpleasant,” and “depressed” scores) were assessed. The depressive mood scores were obtained by combining the last two mood scores [(300 − positive mood) + negative mood]. These mood scores were rescaled in the range 0–100.

#### Assessment of locomotor activity

This watch-type device is also equipped with an activity monitor, which is analogous in performance to the commercial actigraph (Ambulatory Monitors Inc., Ardsley, NY, USA), widely used in clinical fields [24,31,32,47]. The sensor for assessing locomotor activity is a uni-axial piezo-electric accelerometer capable of detecting small changes in wrist acceleration (≥0.01 G/rad/s), which makes it possible to register even slight movements. All subjects were instructed to wear this device on the wrist of their respective non-dominant hand throughout the study period, except while bathing or performing rigorous exercises. Zero-crossing counts were accumulated for every 1-min epoch for the undergraduates and office workers. For adolescents, acceleration counts were accumulated for every 10 seconds; these activity data were aggregated to make 1-min values to ensure compatibility with the other groups. Locomotor activity data for periods in which the subjects were not wearing the device or sleeping were excluded from the analysis. An example of locomotor activity data is shown in Figure 3.

### Data analysis

#### Local statistics of locomotor activity

We calculated the local statistics of locomotor activity data up to the third order moment, but used only the mean and skewness because the first and third order moments are considered to be sufficient to characterize the observed data. While the standard deviation (i.e., the second moment) is a standard measure characterizing variability of given data, it is not appropriate for the case where the data do not obey a normal distribution; the distribution of locomotor activity has nonnegative values, leading to a positively-skewed distribution. Indeed, the standard deviation did not play major roles in predicting depressive mood scores when it was included in the model (results not shown). In contrast, the skewness, as a measure of asymmetry of a distribution, is thought to be more appropriate to characterize the observed asymmetry. Lower or higher mean activity levels could quantify the states related to psychomotor retardation or agitation, respectively [24,26]. Higher skewness could characterize the intermittent bursts of locomotor activity [31,32,48].

In addition, we calculated the skewness from the detrended locomotor activity data, where a trend of activity data is subtracted by fitting the first order polynomial (i.e., linear) function before calculation, This detrending was aimed at eliminating effects of non-stationarity due to, e.g., daily activities; we examined the effects up the third order polynomials without finding systematically different results (results not shown). Thus, we obtained three local statistics of locomotor activity, the mean, skewness, and (linearly) detrended skewness for evaluating the associations with the scores of EMA recordings.

#### Choice of size and location of time frame

The local statistics were calculated from the neighboring data of each EMA within a certain time frame with size N and location K. The value of K (min) represents the central position (location) of the time frame against the timing of EMA recordings, and the size N (min) indicates the data points used to calculate the statistics. Thus, the combination of N and K indicates the time frame starting from N/2 − K points (min) prior to each EMA recording and ending at N/2 + K points (min) after the recording. Therefore, the case where N = 60 and K = −30 indicates the time frame staring from 60 min prior to an EMA recording to the timing of the recording.

The choice of the size of the time frame may be important because they could have a significant impact on the robustness of the statistics and their temporal coincidence with the symptoms. Theoretically, the larger the size of the time frame, the greater is the stability and reliability of the estimates. However, the choice of larger time frames with less localized temporal positions may obscure the information on the momentary associations between locomotor activity and self-reported symptoms. In addition, the choice of the location plays an important role to investigate the casual association, whether locomotor activity precedes or follows changes in momentary symptoms. Thus, careful consideration of such a trade-off is required, while the optimal choice of combination of the size and location is in advance unknown. Therefore, we varied the size N and location K of the time frame to examine their effects.

### Statistical analysis

The data set in the present study has a hierarchical structure, in which EMA recordings were conducted at multiple times during the study period; thus, the recordings and the corresponding local statistics of locomotor activity were nested within the subjects. Therefore, we used multilevel modeling, which is an extension of traditional regression models and has been recommended for the analysis of data with a hierarchical structure including the within- and between-person levels (e.g., individual differences such as age, gender and occupation) [49], using SAS Proc Mixed (SAS 9.2, SAS Institute Inc., Cary, NC). Multilevel modeling can also handle unbalanced data in which the number of the EMA recordings is different across subjects and can include both random and fixed effects together in the same model. A *p* value less than 0.05 was considered significant. For the univariate multilevel modeling, a false discovery rate (FDR) procedure [50] was performed at a *q* value of 0.05 to correct for the multiple comparisons. All models were estimated from the data set sampled from the three groups.

#### Convergent associations among symptoms

To evaluate the convergent associations among self-reported moods and fatigue, simultaneously measured by EMA, we calculated correlations among their scores using 2-level mixed MANOVA models with EMA scores as the dependent variables and the type of symptoms (i.e., depressive mood, anxious mood, and fatigue) as the predictor. Various studies demonstrate the co-variation between depressive and anxious moods, suggesting the possibility to categorize them into the same class of a broader negative affect dimension [51,52,53]. Indeed, the validation study of DAMS confirms the significant positive correlation between these two mood scores [46]. Fatigue is known to have an extremely high comorbidity rate with depressive as well as anxious moods. For example, fatigue is a common complaint in depressive patients [54,55,56,57]. In chronic fatigue syndrome, the high rate of its comorbidity with both moods has been reported [58]. Co-variations of fatigue with these moods have also been reported in patients with cancer [59]. Therefore, these three symptoms would be expected to co-vary with each other if the subjects properly performed the EMA recordings; otherwise, such correlations should be absent.

#### Concurrent associations of symptoms with local statistics of locomotor activity

To investigate the concurrent associations between self-reported symptoms and simultaneous locomotor activity data, the following multilevel models were estimated. In all models, the scores of EMA recordings were treated as the dependent variables, and the corresponding local statistics of locomotor activity were modeled as the predictors. Firstly, we estimated univariate multilevel models to test the simple correlations between the symptoms and local statistics. In addition, we also examined the effects of the choice of time frame to find an optimal combination of size and location for evaluating the local statistics. With regard to the size of the time frame, we considered 12 different conditions: N = 120, 110, 100, …, 20, 10 min. We also considered 25 different locations: K = −60, −55, −50, …, 55, 60 min. We considered a total of 300 combinations (12 sizes × 25 locations) for each symptom and statistic. The FDR with the *q* value of 0.05 was used as the multiple comparison adjustment.

Secondly, all possible multivariate multilevel models were evaluated by considering the three predictors and their interaction terms. However, the terms consisting of both skewness and detrended skewness were not considered; the interaction terms we considered were: mean × skewness and mean × detrended skewness. Furthermore, we considered both fixed (i.e., the slope of the predictor does not vary across individuals) and random (i.e., the slope varies across individual) effects for all predictors. In this report, we focus on the results for depressive mood because we could not find a significant association of locomotor activity with fatigue or anxious mood.

We adopted the following procedures to evaluate the goodness-of-fit of the models. If the inclusion of a random effect into a predictor significantly improved the residual variance of the data explained by the model, we considered the random effect for that term [60]. After determining the type of effect of all predictors for all models, we compared the goodness-of-fit by using two types of tests depending on the structure of the models. While comparing the models with nested or inclusive relations, where all predictors used in one model were a subset of the predictors used in another model, we tested the deviance statistic [61]. This approach tests the difference in the −2 log-likelihood on the basis of the chi-square test with degrees of freedom equal to the difference in the number of model parameters to be estimated. If the difference in the −2 log-likelihood was not significant, the model with fewer parameters was selected as the preferable one on the basis of the *principle of parsimony*. For the non-nested models, we used a statistical test based on the Akaike information criteria (AIC), in which the difference of AIC between models is evaluated using the *z*-test [62].

To determine the best-fitting model for describing the variations of depressive mood scores, we used a time frame of 60 min centered around the EMA recording (i.e., N = 60, K = 0) on the basis of the investigation of univariate multilevel models (see Results). After determining the best-fitting model, we also considered the effects of size and location by varying them. We considered 12 different sizes (N = 120, 110, 100, …, 20, 10) keeping the location of the frame (K = 0). We also considered 13 different locations (K = −30, −25, −20, …, 25, 30) with the size set at 60 min.

#### Cross validation for association of depressive mood with local statistics of locomotor activity

To cross validate the associations of depressive mood scores with local statistics of locomotor activity among the three groups, an ad hoc analysis was performed for the best-fitting model. We added a categorical variable representing the groups (adolescents, undergraduates, and office workers) to the best-fitting model in order to test whether the concurrent associations differed among categories.
